# The Pathologic Findings of Skin, Lymph Node, Liver, and Bone Marrow in Patients With Adult-Onset Still Disease: A Comprehensive Analysis of 40 Cases

**DOI:** 10.1097/MD.0000000000000787

**Published:** 2015-05-01

**Authors:** Hyoun-Ah Kim, Jee Eun Kwon, Hyunee Yim, Chang-Hee Suh, Ju-Yang Jung, Jae Ho Han

**Affiliations:** From the Department of Rheumatology (H-AK, C-HS, J-YJ); and Department of Pathology (JEK, HY, JHH), Ajou University School of Medicine, Suwon, Korea.

## Abstract

Adult-onset Still disease (AOSD) is characterized by fever, skin rash, and lymphadenopathy with leukocytosis and anemia as common laboratory findings. We investigated the characteristic pathologic findings of skin, lymph node, liver, and bone marrow to assist in proper diagnosis of AOSD.

Forty AOSD patients were included in the study. The skin (26 patients), lymph node (8 patients), liver (8 patients), or bone marrow biopsies (22 patients) between 1998 and 2013 were retrospectively analyzed. AOSD patients were diagnosed according to the Yamaguchi criteria after excluding common infections, hematological and autoimmune diseases. Immunohistochemistry, immunofluorescence, and Epstein–Barr virus–encoded RNA (EBER) in situ hybridization were performed.

Most skin biopsies revealed mild lymphocytic or histiocytic infiltration in the upper dermis. Nuclear debris was frequently found in the dermis in 14 cases (53.8%). More than half of the cases (n = 14, 53.8%) showed interstitial mucin deposition. Some cases showed interface dermatitis with keratinocyte necrosis or basal vacuolization (n = 10; 38.5%). The lymph node biopsies showed a paracortical or diffuse hyperplasia pattern with immunoblastic and vascular proliferation. The liver biopsies showed sparse portal and sinusoidal inflammatory cell infiltration. All cases showed various degrees of Kupffer cell hyperplasia. The cellularity of bone marrow varied from 20% to 80%. Myeloid cell hyperplasia was found in 14 out of the 22 cases (63.6%). On immunohistochemistry, the number of CD8-positive lymphocytes was greater than that of CD4-positive lymphocytes in the skin, liver, and bone marrow, but the number of CD4-positive lymphocytes was greater than that of CD8-positive lymphocytes in the lymph nodes.

The relatively specific findings with respect to the cutaneous manifestation of AOSD were mild inflammatory cell infiltration in the upper dermis, basal vacuolization, keratinocyte necrosis, presence of karyorrhexis, and mucin in the dermis. In all cases, pathologic findings in the lymph nodes included paracortical hyperplasia with vascular and immunoblastic proliferation. Skin and lymph node pathology in addition to clinical findings can aid in the diagnosis of AOSD.

## INTRODUCTION

Adult-onset Still disease (AOSD) is an acute, systemic inflammatory disorder of unknown etiology. It is clinically characterized by high spiking fever, arthralgia, typical evanescent skin rash, lymphadenopathy, and hepatosplenomegaly.^1,2^ Leukocytosis, anemia, thrombocytosis, and elevated acute phase reactants are the common abnormal laboratory findings in AOSD. However, these clinical features and laboratory results are nonspecific, and they overlap with those of autoimmune disease, infections, and hematologic malignancies.^3,4^ Therefore, the spectrum of differential diagnosis is wide and may cause difficulty in making the correct diagnosis. Pathologic confirmation of affected organs is occasionally needed for accurate diagnosis, as well as for ruling out malignancies.

The typical Still rash is an evanescent, salmon-pink, macular, or maculopapular eruption. The rash is a well-accepted major diagnostic criteria of AOSD.^2,4^ In addition to the typical rash, an atypical, nonevanescent rash including pruritic persistent papules or plaques has been reported in active AOSD.^5–9^ However, the skin lesions are often misdiagnosed as an allergic reaction to drugs, such as nonsteroidal anti-inflammatory drugs or antibiotics, and therefore, the diagnosis of AOSD could be delayed.^10^ The cutaneous pathology described previously is nonspecific.^5,6,8,11,12^ Lymphadenopathy may develop in 44% to 90% patients with AOSD and it causes difficulty in the differential diagnosis from hematologic malignancies.^13–15^ In contrast to the nonspecific cutaneous pathology, characteristic lymph node histology of AOSD has been described in several reports.^16–19^ The other clinical manifestations are related to hepatic dysfunction and hematologic abnormalities. However, there are limited reports on liver and bone marrow pathology in patients with AOSD; the histopathologic findings of these organs in AOSD are not well established.^4,20–23^ Therefore, we characterized the pathologic findings of skin, lymph node, liver, and bone marrow in patients with AOSD to assist in the proper diagnosis of AOSD.

## METHODS

### Subjects

Forty AOSD patients were included in the study. AOSD patients who had received skin, lymph node, liver, or bone marrow biopsies between 1998 and 2013 were retrospectively analyzed. AOSD patients were diagnosed according to the Yamaguchi criteria, after excluding common infections, hematological and autoimmune diseases.^2^

Medical history, clinical symptoms, and information on physical examinations were entered into a database. Each patient underwent a series of laboratory tests, including complete blood count (CBC), erythrocyte sedimentation rate (ESR), C-reactive protein (CRP), rheumatoid factor (RF), antinuclear antibody (ANA), ferritin, and liver function tests. AOSD activity was evaluated according to the method described by Pouchot et al,^3^ which assigns a score from 0 to 12 and adds 1 point for each of the following manifestations: fever, typical rash, pleuritis, pneumonia, pericarditis, hepatomegaly or abnormal liver function tests, splenomegaly, lymphadenopathy, leukocytosis ≥15,000/mm^2^, sore throat, myalgia, and abdominal pain. This study was approved by the Institutional Review Board of our hospital.

### Histopathologic Interpretation and Analysis

Skin biopsies were obtained from 26 patients, lymph node biopsies from 8 patients, liver biopsies from 8 patients, and bone marrow biopsies from 22 patients. We evaluated the hematoxylin and eosin-stained sections from the skin, lymph node, liver, and bone marrow biopsies. The Wright–Giemsa stained bone marrow aspirates were also examined. The slides were independently examined by 3 observers (J.H.H., J.E.K., and H.Y.) with respect to the following skin histologic parameters: epidermal changes, degree of dermal lymphocytic or histiocytic infiltration, and presence of karyorrhexis, vasculitis, and interstitial mucin. Lymph node histologic parameters were assessed in terms of different patterns of reaction (follicular, paracortical, or diffuse hyperplasia), types of infiltrating inflammatory cells, degree of immunoblast proliferation, and degree of vascular proliferation. Liver histologic parameters were examined in terms of portal inflammation, sinusoidal inflammation, and Kupffer cell hyperplasia. We graded the pathologic parameters into absent (0), mild (1), moderate (2), or severe (3). The marrow was evaluated with regard to M/E ratio, cellularity, and relative count of plasma cells. The marrow was considered hyperplastic when the cellularity was ≥70%. Immunohistochemistry and in situ hybridization for the detection of Epstein–Barr virus–encoded RNA (EBER) were performed on the formalin-fixed, paraffin-embedded sections. The antibody panel included CD4 (1:10 dilution; Thermo Fisher Scientific, Fremont, CA), CD8 (1:50 dilution; Thermo Fisher Scientific), and CD68 (1:200 dilution; Novocastra Laboratories Ltd, Newcastle, UK). Each marker was quantified based on the extent of antibody staining. The percentage of positive inflammatory cells was graded on a scale from 1 to 3: 0, none; 1, 0 to 1/3; 2, 1/3 to 2/3; 3, 2/3 to 1 in skin, lymph node and liver, and 1,1% to 10%; 2, 11% to 50%; 3, 51% to 100% among mononuclear cells in bone marrow. In situ hybridization for the detection of EBER was performed by first applying the Epstein–Barr virus probe (Novocastra Laboratories Ltd) that contains a random mixture of fluorescein isothiocyanate (FITC)-labeled oligonucleotides that hybridize to EBER1 and EBER2. An anti-FITC antibody was subsequently added and detection was performed with the Ultratech HRP streptavidin-biotin universal detection system (Immunotech, Marseille, France). The data on immunofluorescence staining of 5-μm-thick frozen tissue sections were available in 11 cases of skin biopsies. The panel included polyclonal antibodies to IgG (1:40 dilution; DAKO, Glostrup, Denmark), IgA (1:40 dilution; DAKO), IgM (1:40 dilution; DAKO), C3 (1:40 dilution; DAKO), and fibrinogen (1:40 dilution, DAKO).

### Statistical Analyses

All data are shown as means ± standard deviations (SDs) or number (%). All statistical analyses were performed using SPSS version 20.0 (SPSS, Chicago, IL). The Mann-Whitney U-test and the χ^2^ test were used to compare frequency and categorical data between typical and atypical cutaneous manifestations. The comparison of prognosis between the patients with hemophagocytic features and those without hemophagocytic features was performed using the χ^2^ test. A *P* value < 0.05 was regarded as statistically significant.

## RESULTS

### Clinical Characteristics of 40 AOSD Patients

Table 1 summarizes the clinical characteristics of 40 AOSD patients and the biopsy type. The mean age of the AOSD patients was 43.4 ± 16.9 years and women comprised 70%. The main clinical symptoms included high spiking fever (97.5%), skin rash (87.5%), arthritis (67.5%), sore throat (47.5%), and splenomegaly (45%). Almost all skin manifestations were maculopapular eruptions on the trunk, upper and lower extremities (n = 17, 65.4%). Five patients (19.2%) had persistent pruritic eruptions, and 2 patients had painful swelling of the low extremities. Two patients had papulopustular lesions on the trunk. An elevated ESR >20 mm/h was observed in 40 patients (100%), an elevated CRP >0.8 mg/dL was observed in 40 patients (100%), leukocytosis ≥10,000/mm^3^ was observed in 26 patients (65%), and elevated ferritin levels ≥10,000 ng/mL were observed in 16 patients (40%).

**TABLE 1:**
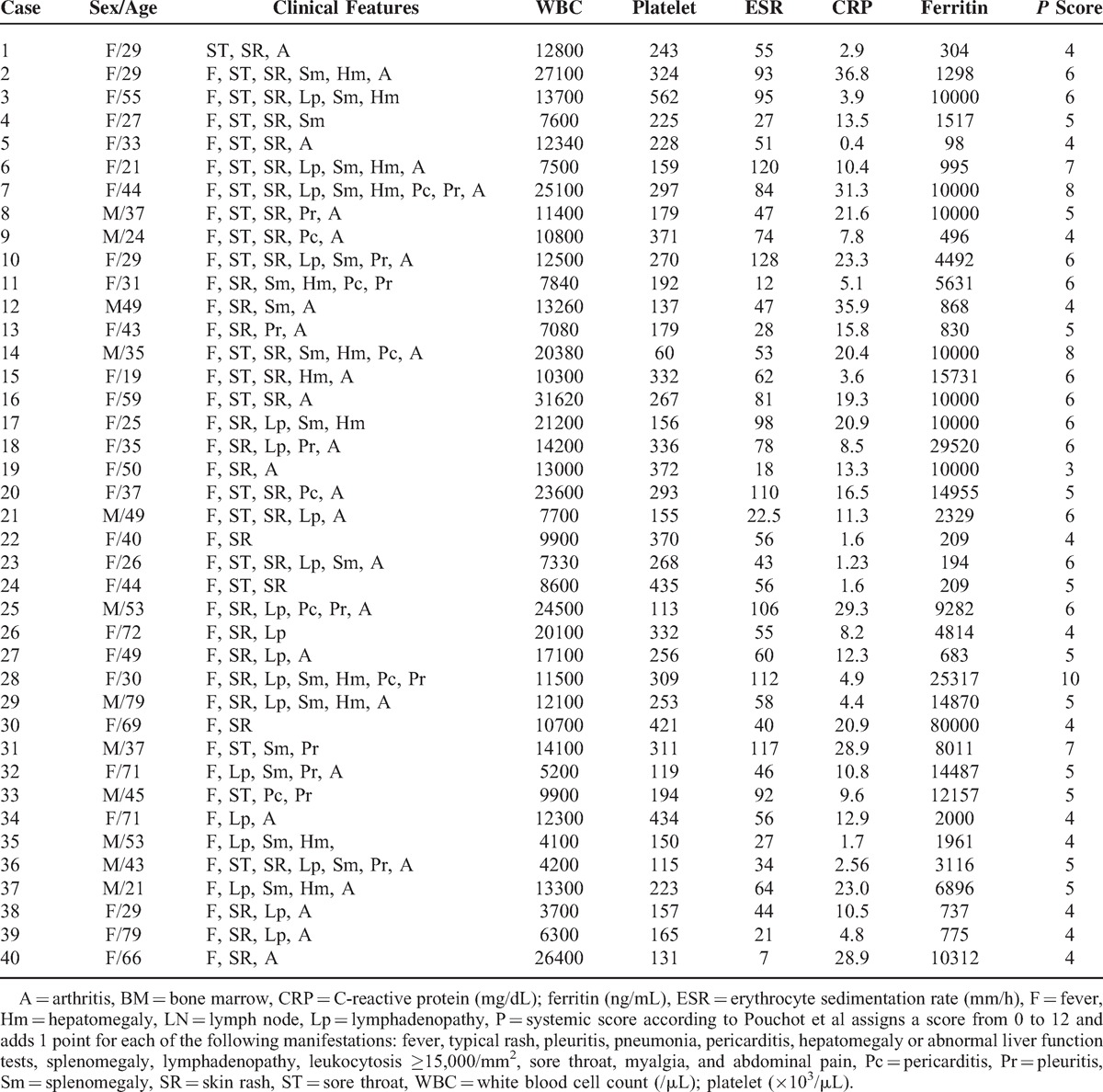
Clinical Characteristics of Adult-Onset Still Disease Patients Who Received Biopsy of Skin, Lymph Node, Liver, or Bone Marrow

### Skin Pathologic Findings

**FIGURE 1:**
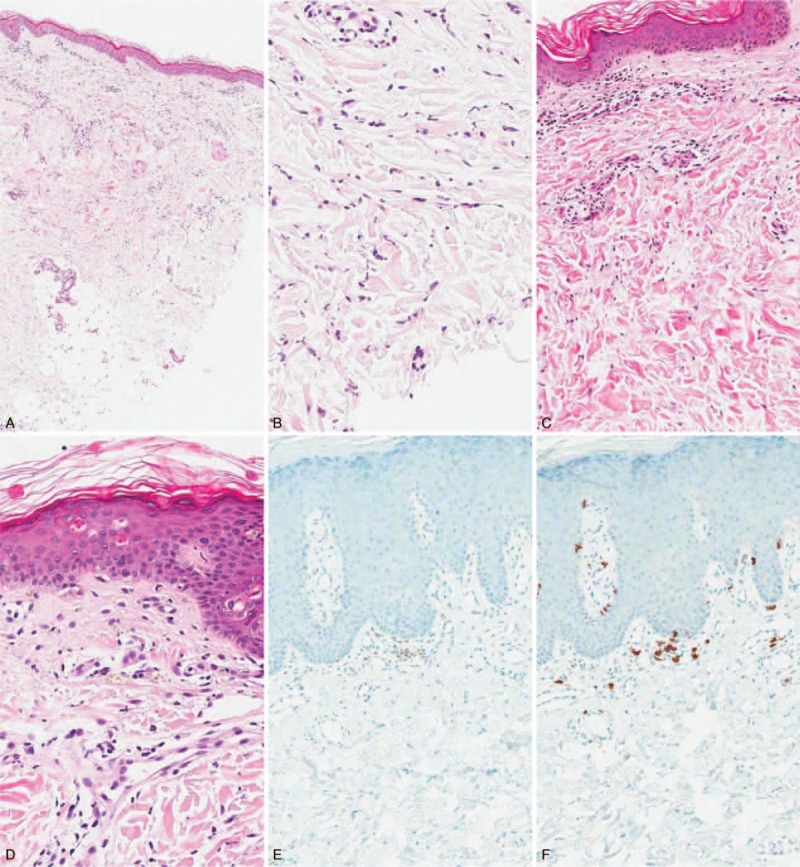
Cutaneous findings in patients with adult-onset Still disease. Biopsy in case no. 24 shows mild perivascular inflammatory cell infiltration (A) with karyorrhexis (B). Biopsy in case no. 18 shows dermal mucin deposition that splayed the dermal collagen fibers (C). Biopsy in case no. 3 shows mild lymphohistiocytic infiltration with karyorrhexis and a few necrotic keratinocytes in the epidermis (D). Immunohistochemial staining for CD4 (E) and CD8 (F) in case no. 17. The number of CD8-positive lymphocytes is greater than that of CD4-positive lymphocytes. (Original magnification, ×40 (A), ×100 (C, E, F), ×200 (B, D).

Table 2 and Figure 1 show the histopathologic features with respect to cutaneous manifestations of AOSD. The skin biopsies showed mild lymphohistiocytic infiltration in the upper dermis. The inflammatory cell infiltrates extended into the subcutaneous fat tissue in 4 cases (15.4%). Nuclear debris was found in the dermis in 14 cases (53.8%). Four cases had parakeratosis; however, the degree of parakeratosis was minimal and negligible. More than half of the cases (n = 14, 53.8%) showed interstitial mucin deposition. A few scattered neutrophils were noted in 7 cases (26.9%), and scattered eosinophils and plasma cells were noted in only 1 case (case no. 14). Case nos. 12 and 18 showed extravasation of red blood cells. Vasculitis was seen in only 2 cases (7.7%). Epidermal changes, such as an interface dermatitis with basal vacuolization or a few necrotic keratinocytes, were observed in some cases (n = 10; 38.5%).

**TABLE 2:**
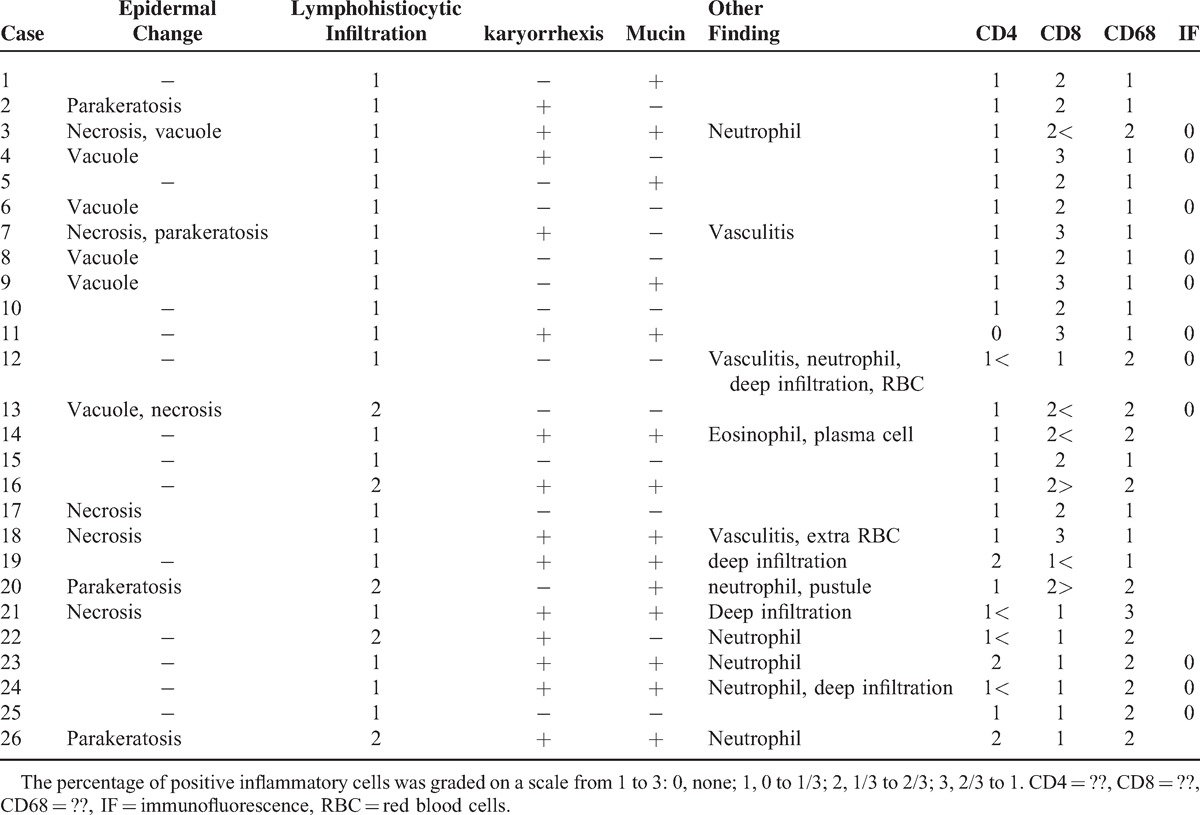
Histopathologic and Immunohistochemical Staining Features With Respect to Cutaneous Manifestation of Adult-Onset Still Disease

Most of the infiltrating cells were positive for CD68 and CD8 on immunohistochemistry. The percentage and intensity of staining of the positive cells were greater for CD8 than CD68 (57.7%). The number of CD8-positive lymphocytes was greater than that of CD4-positive lymphocytes in 23 (88.5%) out of the 26 cases. Direct immunofluorescence staining was negative in a total of 11 cases.

### Lymph Node Pathologic Findings

**FIGURE 2:**
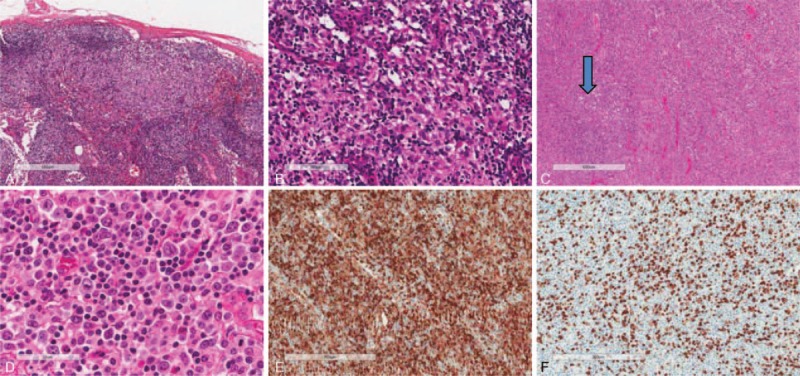
Lymph node findings in patients with adult-onset Still disease. Biopsy case no. 3 shows nodular expansion of the paracortex (A) by pale-staining histiocytes, dendritic cells, or Langerhans cells (B). Biopsy of case 4 shows paracortical or diffuse hyperplasia with vascular hyperplasia (C). A residual lymphoid follicle is seen (arrow). The paracortex is composed of large immunoblasts and small lymphocytes with occasional eosinophils (D). Immunohistochemical staining for CD4 (E) and CD8 (F) in case no. 4. The number of CD4-positive lymphoid cells is greater than that of CD8-positive lymphoid cells. (Original magnification, ×40 (A, C), ×100 (E, F), ×200 (B), ×400 (D).

Table 3 and Figure 2 show the histopathologic and immunohistochemical staining features with respect to lymph node manifestations of AOSD. Five cases showed only paracortical hyperplasia, and 3 cases showed a mixed pattern; paracortical and diffuse (case nos. 4 and 29) or paracortical, follicular, and diffuse (case no. 6). Vascular proliferation was moderate to severe in all cases, and immunoblastic proliferation was moderate to severe in 5 out of the 8 cases (62.5%). Two cases showed mixed inflammatory cell infiltrations with scattered neutrophils or eosinophils. Case no. 27 had pericapsular endarteritis, and case no. 29 showed hemophagocytic features with a positive signal for EBER in a few scattered small lymphocytes. Pathologic findings of case nos. 3 and 17 were similar to those of dermatopathic lymphadenitis (Figure 2A and B), and pathologic findings of case nos. 4, 27, and 29 were similar to those of angioimmunoblastic T-cell lymphoma (AITL) (Figure 2C and D). The number of CD4-positive lymphocytes was greater than that of CD8-positive lymphocytes in 7 out of the 8 cases (87.5%) on immunohistochemistry (Figure 2E and F).

**TABLE 3:**
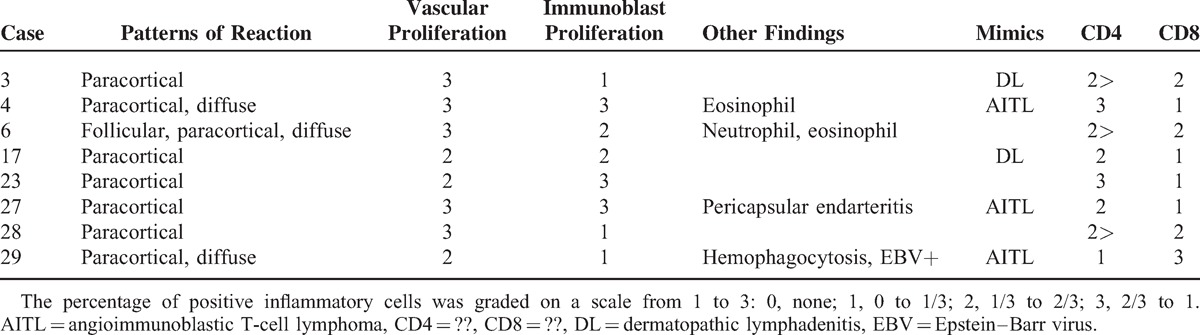
Histopathologic and Immunohistochemical Staining Features of Lymph Node in Patients With Adult-Onset Still Disease

### Liver Pathologic Findings

Liver biopsies showed mild portal and sinusoidal inflammatory cell infiltration (Table 4). Lymphocytic infiltration was mainly observed in the portal area, and neutrophil infiltration was found in the sinusoids. A few scattered sinusoidal eosinophils were noted in case no. 8. All cases showed various degrees of Kupffer cell hyperplasia. Four cases (50%) showed mild Kupffer cell hyperplasia, and the remaining cases showed moderate to severe Kupffer cell hyperplasia. Case no. 4 showed peliosis hepatitis, and case nos. 8, 15, and 30 showed hemophagocytic features. Case no. 30 showed hepatocyte necrosis and portal fibrosis. The number of CD8-positive lymphocytes was greater than that of CD4-positive lymphocytes in 7 out of the 8 cases (87.5%) on immunohistochemistry.

**TABLE 4:**
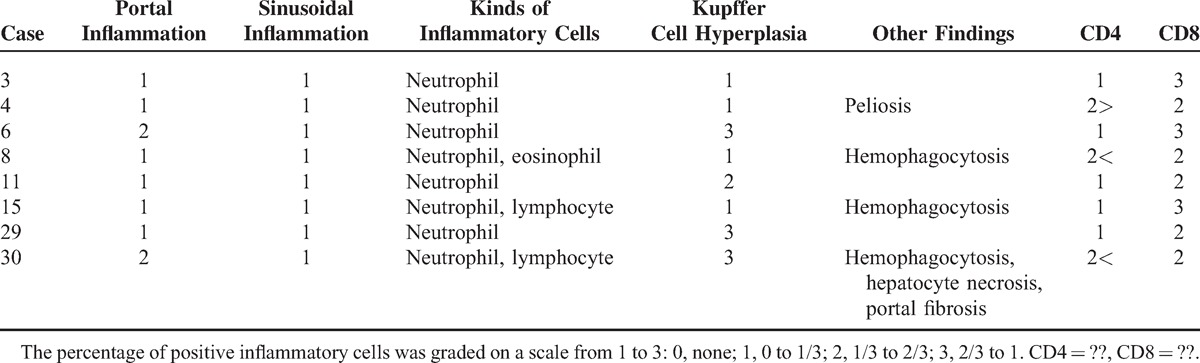
Histopathologic and Immunohistochemical Staining Features With Respect to Liver Manifestation of Adult-Onset Still Disease

### Bone Marrow Pathologic Findings

The cellularity of bone marrow varied from 20% to 80% (Table 5). Hyperplastic bone marrow was observed in 7 out of the 22 cases (31.8%). Fourteen cases showed normal cellularity, and only 1 case (case no. 33) had hypocellular bone marrow. Myeloid cell hyperplasia was found in 14 out of the 22 cases (63.6%). The plasma cell fraction was in the normal range. Four cases showed hemophagocytic features, and 2 cases had positive signal for EBER among the 6 cases. Immunohistochemistry showed that the number of CD8-positive lymphocytes was greater than that of CD4-positive lymphocytes in 20 out of the 22 cases (90.9%).

**TABLE 5:**
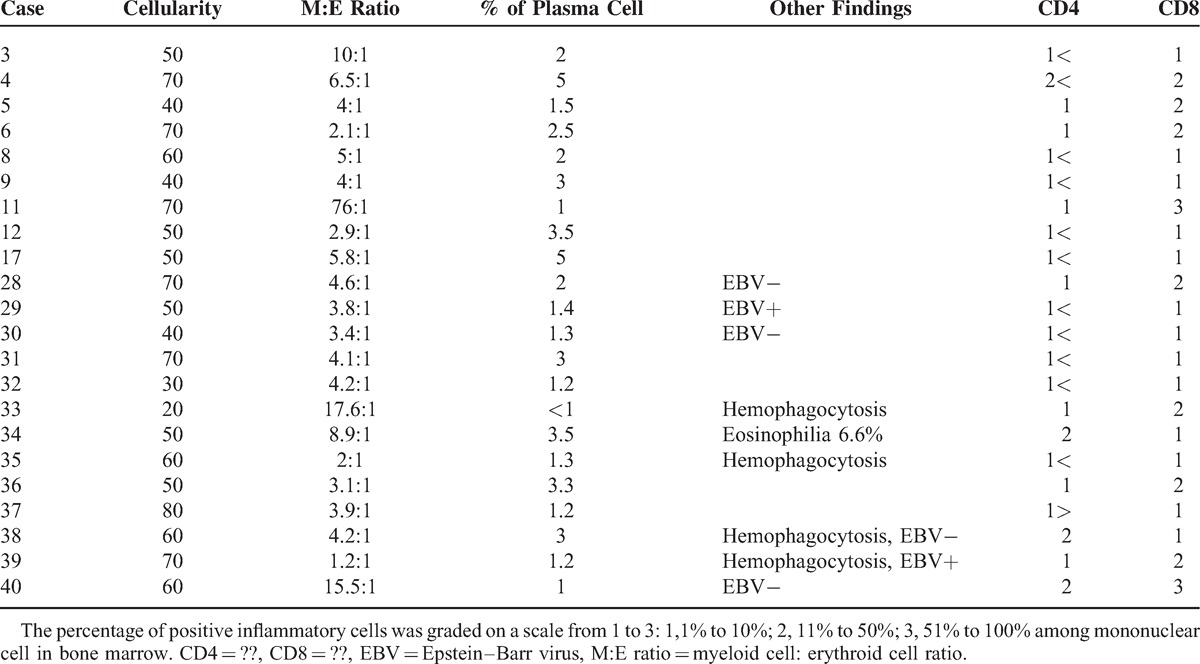
Histopathologic and Immunohistochemical Staining Features With Respect to Bone Marrow Manifestation of Adult-Onset Still Disease

## DISCUSSION

We described the clinical and histopathologic findings of the involved skin, lymph node, liver, and bone marrow in 40 patients with AOSD in order to assist in proper diagnosis of AOSD in affected organs.

Cutaneous manifestations of AOSD are common, and they can be variable. However, the most common finding is evanescent macular or maculopapular eruptions.^1,2^ Atypical or persistent skin lesions in AOSD have also been reported in case reports or small case series.^5–9,12,24^ The evanescent macular or maculopapular eruptions were the most common finding in this study (65.4%), and atypical skin lesions including persistent pruritic eruptions were observed in 9 patients (34.6%). Histology of the typical Still rash is characterized by a relatively sparse perivascular mixed inflammatory infiltrate containing some neutrophils.^2^ In a recent study, persistent pruritic eruptions revealed solitary or cluster necrotic keratinocytes in the superficial epidermis, with infiltration of lymphocytes and neutrophils in the upper and mid dermis.^9^ Vacuolization and mucin deposition have also been described in the literature.^9,11,12,25^ The histologic findings with respect to the cutaneous manifestations in this study were variable, and there were no histopathologic differences between typical and atypical skin eruptions (data not shown). The relatively common features were mild lymphocytic and histiocytic infiltration in the upper dermis, presence of nuclear debris (53.8%), interstitial mucin deposition (53.8%), basal vacuolization, and keratinocyte necrosis. However, these histologic findings and clinical features including rash, fever, and lymphadenopathy are shared with systemic lupus erythematosus (SLE) and Kikuchi disease.^26^ Nevertheless, lupus patients could have different laboratory results including autoantibodies, hypocomplementemia, and leukopenia, and their cutaneous histopathologic findings reveal more dense inflammatory cell infiltration, mostly CD4-positive lymphocyte, and positive immunofluorescence results. It is unclear why the number of CD8-positive lymphocytes was greater than that of CD4-positive lymphocytes in skin biopsy of the AOSD patient. However, predominance of CD8-positive lymphocytes over CD4-positive lymphocytes was related to a direct cytotoxic immune reaction in the pathogenesis of interface dermatitis or karyorrhexis. The cutaneous histopathology in Kikuchi disease shows predominant histiocytic infiltration, frequent nuclear debris, and absence of infiltrating neutrophils. Additionally, the lymph node histopathology in Kikuchi disease and SLE reveals histiocytic necrotizing lymphadenitis, which is different from the lymph node pathology in AOSD.

Although lymphadenopathy is a common manifestation of AOSD, there are only a few reports of lymph node histopathology, and the majority of them are case reports.^16–19,27^ Nevertheless, proper understanding of lymph node histopathology is very important, because the clinical manifestations of AOSD, such as fever, lymphadenopathy, and hepatosplenomegaly, are similar to those of malignant lymphoma. Initially, the histopathologic finding of lymph nodes in AOSD was described as nonspecific reactive hyperplasia.^28^ Subsequently, pathologic findings of lymph nodes were described as plasma cell and neutrophil infiltrates with signs of reactive hyperplasia, follicular pattern of reactive hyperplasia, or atypical paracortical hyperplasia in AOSD.^13,16^ One study analyzed histopathologic patterns in 12 patients with AOSD, and reported that lymph node lesions in AOSD have a dynamic histologic spectrum, including atypical paracortical hyperplasia, burned out histiocytic reaction, exuberant immunoblastic reaction, and follicular hyperplasia.^19^ The consistent finding of AOSD lymphadenopathy in this study was paracortical hyperplasia accompanied by vascular and immunoblastic proliferations. Initially, we considered the possibility of AITL in 3 cases. AITL typically presents with generalized lymphadenopathy, hepatosplenomegaly, and systemic symptoms. Other common findings are skin rash, arthritis, pleural effusion, and ascites. In addition, both paracortical and immunoblastic proliferation can be confused with AITL. The histopathologic differences between these conditions are as follows: first, the neoplastic T-cells in AITL are medium to large lymphoid cells with abundant clear cytoplasm; however, this feature was not observed in our 3 cases. Second, we could not detect EBER-positive immunoblast and proliferation of CD21-positive irregular follicular dendritic cells, which are characteristics of AITL. Third, 3 cases that mimicked AITL showed absence of follicular helper T-cell markers, including PD-1, BCL-6, CD10, and CXCL13 (data not shown).

Liver enzyme elevation or hepatomegaly is frequently observed in patients with AOSD, but they are usually mild and transient.^4^ Liver injury has been attributed to drug allergies or toxicities, but it also occurs in AOSD patients who are not exposed to drugs. Liver histopathologic findings include inflammatory cells infiltration of the portal areas, focal hepatitis with necrosis, mild chronic necroinflammatory changes, mild interstitial hepatitis, and mild portal fibrosis.^2,4,29–32^ These changes that are features of nonspecific reactive hepatitis were similar to those in our study. However, Kupffer cell hyperplasia with hemophagocytic features requires further attention. Hemophagocytic features were also seen in the lymph node and bone marrow. A previous report showed reactive hemophagocytosis in 16.7% (2/12) of bone marrow biopsies.^23^ Kumakura et al^33^ suggested that pancytopenia may be absent in patients with AOSD complicated with the hemophagocytic syndrome. Eight patients had hemophagocytic features (32%) among the study patients who received lymph node, liver, or bone marrow biopsies (n = 25). These patients did not have peripheral cytopenia, and the diagnosis of hemophagocytic lymphohistiocytosis (HLH) could be excluded according to the HLH-2004: diagnostic and therapeutic guidelines for HLH.^34^ The prognosis of the patients with hemophagocytic features (none of the patients died) in biopsy results was not poor, as compared with that of the patients without hemophagocytosis (3 patient died, *P* = 0.296). These results suggested that physicians may have treated the patients enthusiastically when the patient had hemophagocytic features in the biopsy results. However, the number of cases was relatively small, and this finding could be due to selection bias.

A common hematologic feature is leukocytosis with neutrophilia, and it is accepted as one of the major diagnostic criteria of AOSD.^4^ Several mechanisms have been suggested for neutrophilia in AOSD, such as increased stimulation for release from the marginating leukocyte pool, prolongation of the peripheral half-life, and increased stimulation of myelopoiesis.^23^ This hypothesis was supported by findings of hypercellularity with myeloid hyperplasia, which is similar to our results.

In conclusion, the relatively specific findings with respect to the cutaneous manifestations were mild inflammatory cell infiltration in the upper dermis, vacuolization in the basal layer of epidermis, keratinocyte necrosis, presence of karyorrhexis, and mucin in the dermis. In all cases, pathologic findings in the lymph nodes included paracortical hyperplasia with vascular and immunoblastic proliferation, which mimicked AITL. The pathologic findings of liver and bone marrow were rather nonspecific except for the hemophagocytic features. It is important to recognize the cutaneous and lymph node pathology in AOSD patients, and we can suspect AOSD in cases showing the aforementioned histopathologic findings and clinical manifestations.
